# One-pot synthesis of S-scheme MoS_2_/g-C_3_N_4_ heterojunction as effective visible light photocatalyst

**DOI:** 10.1038/s41598-021-94129-0

**Published:** 2021-07-20

**Authors:** Ha Tran Huu, My Duyen Nguyen Thi, Van Phuc Nguyen, Lan Nguyen Thi, Thi Thuy Trang Phan, Quoc Dat Hoang, Huy Hoang Luc, Sung Jin Kim, Vien Vo

**Affiliations:** 1 Faculty of Natural Sciences, Quy Nhon University, 170 An Duong Vuong, Quy Nhon, 55000 Binh Dinh Vietnam; 2 Vietnam Ministry of Science and Technology, 113 Tran Duy Hung, Cau Giay, Hanoi, 10000 Vietnam; 3 Faculty of Physics, Hanoi National University of Education, Hanoi, 100000 Vietnam; 4 Department of Chemistry and Nano Science, Ewha Womans University, Seoul, 120-750 South Korea

**Keywords:** Photocatalysis, Pollution remediation

## Abstract

Despite pioneering as the holy grail in photocatalysts, abundant reports have demonstrated that g-C_3_N_4_ performs poor photocatalytic activity due to its high recombination rate of photo-induced charge carriers. Many efforts have been conducted to overcome this limitation in which the semiconductor–semiconductor coupling strategies toward heterojunction formation were considered as the easiest but the most effective method. Herein, a one-pot solid-state reaction of thiourea and sodium molybdate as precursors at different temperatures under N_2_ gas was applied for preparing composites of MoS_2_/g-C_3_N_4_. The physicochemical characterization of the final products determines the variation in contents of components (MoS_2_ and g-C_3_N_4_) via the increase of synthesis temperature. The enhanced photocatalytic activity of the MoS_2_/g-C_3_N_4_ composites was evaluated by the degradation of Rhodamine B in an aqueous solution under visible light. Therein, composites synthesized at 500 °C showed the best photocatalytic performance with a degradation efficiency of 90%, much higher than that of single g-C_3_N_4_. The significant improvement in photocatalytic performance is attributed to the enhancement in light-harvesting and extension in photo-induced charge carriers’ lifetime of composites which are originated from the synergic effect between the components. Besides, the photocatalytic mechanism is demonstrated to well-fit into the S-scheme pathway with apparent evidences.

## Introduction

Recently, among advanced oxidation processes applied for organic pollutant degradation, photocatalysis has attracted much attention due to its utilization of solar radiation as green energy source and oxygen in air as abundant oxidant^1^. Consequently, various available oxide semiconductors such as TiO_2_, ZnO, SnO_2_, etc., have been widely investigated^2–4^. However, beside their advantages in low economic and environmental cost, suitable redox potential^2^, the weaknesses, such as being mainly activated by ultraviolet irradiation and high rate of recombination of photo-induced charge carriers, limit their practical application^2–4^. Therefore, it is necessary for searching alternatives with reasonable band structure for acceptable photocatalytic performance in visible light region.

Since its first time reported in 2009^5^, carbon nitride with graphite-like structure (g-C_3_N_4_), a metal-free organic semiconductor, has allured significant attention as a potential photocatalyst due to its moderate band gap of 2.7 eV, relatively chemical stability and thermal stability up to 600 °C^5^. Although light harvesting ability of g-C_3_N_4_ shifts toward visible region, photocatalytic performance of pure g-C_3_N_4_ is insufficient owning to its relatively low light absorption coefficient, short lifetime of photo-generated electrons and holes, and low specific surface area^6,7^. In order to overcome these disadvantages, various methods have been investigated such as co-polymeration^8^, altering different precursors^9^, nonmetal doping^7,10,11^, and exfoliation into thin layers^12^. In addition to these methods, constructing g-C_3_N_4_ with other semiconductors in heterojunction has been considered as an efficient solution to improve the separation of photo-generated electron–hole pairs^13–15^.

Molybdenum disulfide (MoS_2_), a typical 2D layered material, has been widely investigated in potential applications such as lithium ion batteries, electronics, optoelectronics, supercapacitors and sensors^16–18^. Moreover, with a unique electron structure and band gap of 1.3 eV, it has also attracted as a co-photocatalyst in the water splitting^19^, and the degradation of organic pollutants in aqueous solution^20–22^. These reports showed that the presence of MoS_2_ is beneficial in improvement of light harvesting, electron transfer at the interfaces, and charge carriers separation in the composite materials. These benefits may come from synergistic effect between two semiconductors based on their adequate band positions and good lattice matching^23^. Therefore, coupling MoS_2_ to g-C_3_N_4_ to form a reasonable heterojunction is a suitable strategy toward a high photocatalytic performance composite.

To our knowledge, the preparation of MoS_2_/g-C_3_N_4_ composites was reported in several ways: (1) grafting two available components of MoS_2_ and g-C_3_N_4_ in water or organic solvents assisted with sonicated or hydrothermal treatment^24–26^; (2) formation of MoS_2_ in the presence of g-C_3_N_4_^27,28^, (3) formation of g-C_3_N_4_ in the presence of MoS_2_^29,30^. However, there have been few reports in which MoS_2_ and g-C_3_N_4_ are formed at the same time. In fact, this strategy is facile, scalable and eco-friendly^31^. In this work, MoS_2_/g-C_3_N_4_ composites were prepared via a facile one-step process, in which mixtures of the two precursors, thiourea and sodium molybdate, were calcined in N_2_ gas flow at different temperatures. During synthesis, g-C_3_N_4_ offers as a buffering media to exfoliate MoS_2_, while, in turn, MoS_2_ accelerates the decomposition of g-C_3_N_4_ at high temperature toward N-deficiency form with enhanced charge transport. Photocatalytic performance of the composites was evaluated by degradation of Rhodamine B (RhB) under visible light.

## Results

### Characterization of the materials

**Figure 1:**
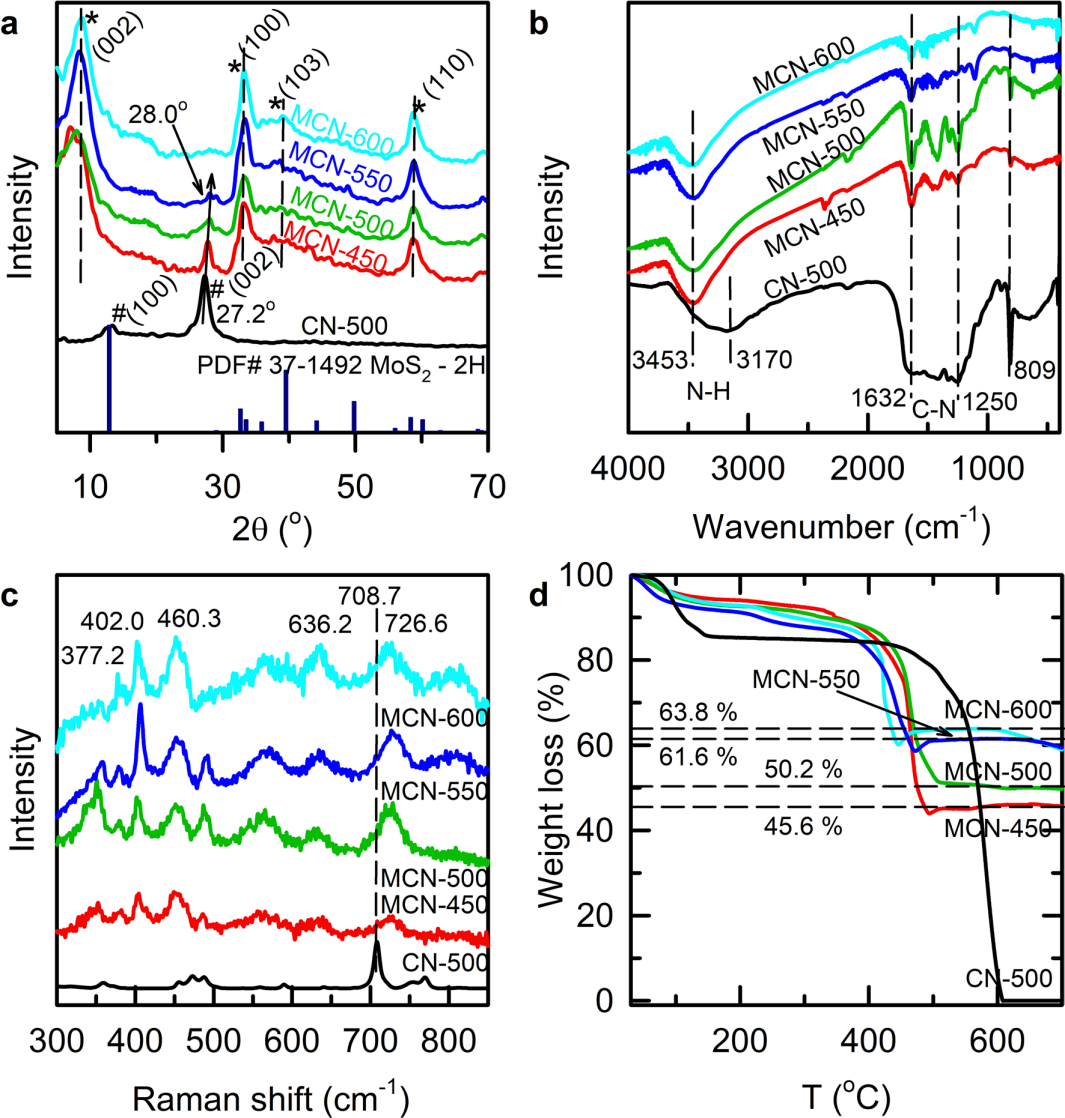
(**a**) XRD patterns; (**b**) FT-IR spectra; (**c**) Raman spectra; and (**d**) TG–DTA curves of CN-500, and MCN-T (T = 450, 500, 550, and 600).

The X-ray diffraction (XRD) patterns of the as-prepared samples were presented in Fig. 1a. For the MCN-600 sample, the pattern showed peaks at angles of 2θ = 32.8°; 39.0° and 58.6°, which can be indexed to the (100), (103) and (110) planes, respectively, corresponding to the hexagonal phase of MoS_2_ (PDF#37-1492)^27,32,33^. The CN-500 pattern exhibited two distinct diffraction peaks with a weak one at 13.2° and a strong one at 27.2°, corresponding to the tight interplanar stacking of the aromatic planes in g-C_3_N_4_ and the (002) plane of graphitic materials^27,33^. For the MCN-450, MCN-500 and MCN-550 composites with different synthesis temperatures, the simultaneous presence of the two distinct diffraction peak systems corresponding to the two components of MoS_2_ and g-C_3_N_4_ can be observed, which confirms the formation of MoS_2_/g-C_3_N_4_ heterostructure via a one-step solid-state reaction. In addition, it is observable that as increasing process temperature, there is a reduction in intensity of (002) peaks of g-C_3_N_4_ which illustrates for the decrease in content of this component at higher temperature treatment. For MCN-600, the presence of characteristic peaks for MoS_2_ can be clearly observed, while the g-C_3_N_4_ peak around 27.2° mostly disappeared, which indicates that the MCN-600 mainly consists of MoS_2_. Figure 1a also showed that the peaks corresponding to (002) plane of g-C_3_N_4_ in the composites MCN-450, MCN-500 and MCN-550 shift to diffraction angle (2θ) of 28.0° compared with pure g-C_3_N_4_ (CN-500). This means that there is a reduction in interlayer distance, indicating more dense packing of the g-C_3_N_4_ layers in the composites^34^. Furthermore, the peak at the lowest diffraction angle, which is ascribed to the (002) plane of MoS_2_, in all of the composites, shifted to lower 2θ-range. This illustrates that the stacking interlayer spacing of (002) plane of MoS_2_ in composites was expanded. In fact, using the Bragg equation, the interlayer space of (002), corresponding to the diffraction peak at 2θ of 8.7°, was calculated as 1.01 nm, which is much larger than the theoretical value of 0.62 nm. The expansion of interlayer distance is favorable for photocatalytic process due to offering more active sites^35^.

The proof on characteristic bonding vibrations of components in the composites was identified by Fourier transform infrared (FT-IR) spectroscopy (Fig. 1b). For the CN-500 sample, the peaks at 809 cm^−1^, and in the range of 1250–1632 cm^−1^ are attributed to the breathing mode of tri-s-triazine units and stretching modes of C–N and C=N bonds in aromatic rings of g-C_3_N_4_^24,33–35^. These peaks can be clearly observed in MCN-450 and MCN-500, but diminished in MCN-550 and MCN-600, which is attributed to further polycondensation and partial thermal decomposition with calcination temperature. Additionally, the broad peaks centered at 3453 cm^−1^ are ascribed to the N–H stretching from terminal amino groups and the O–H bond from adsorbed H_2_O^34^. The intensity of these peaks reduces with the increase in calcination temperature, which may be due to higher degree of polycondensation leading to decreasing terminal amino groups.

For more clear observation, the enlarged FTIR spectra in region of 400–600 cm^−1^ were presented in Supplementary Fig. 1. It can be seen that the peak at 419 cm^−1^ corresponding to typical mode of Mo–S bond^24^ can be observed for all the composites. The intensity of this peak increases with treating temperatures which confirmed the supposition that the higher temperature reinforces the MoS_2_ content.

In order to clarify the presence of components in the materials, Raman analysis was conducted and the results were shown in Fig. 1c, which confirms the formation of MoS_2_ and g-C_3_N_4_ phase. All the samples exhibited peaks at 377.2 cm^−1^ (E^1^_2g_) and 402.0 cm^−1^ (A_1g_) that are due to the first-order Raman vibration modes within the S–Mo–S layer^36^. In addition, the variation of board peaks at 460.3 and 636.2 cm^−1^ via change of synthesis temperature could illustrate for the exfoliation degree of MoS_2_ in composites. In fact, the former peak could be deconvoluted into two components: (1) the lower energy component is ascribed to a combined *2LA*(*M*) process^37^; (2) the higher energy counterpart is proposed to the combined of *E*_*1g*_*(M)* + *XA(M)* process^38^. The latter peak at 636.2 cm^−1^ could also be contributed from two components including *E*_*1g*_*(M)* + *2LA(M)* mode (for high-energy part) and *A*_*1g*_*(M)* + *LA(M)* process (for low-energy component)^38^. As shown in Fig. 1c, the evolution of these peaks via the increase of synthesis temperature is characterized for the variation of exfoliation from few-layer (weak signal) to bulk form (strong intensity) of MoS_2_ which is consistent with the observation in XRD^38^. The higher exfoliation is supposed to create more edge sites as well as expose more active centers of MoS_2_ which is beneficial for enhancement in photocatalytic performance^35^. Besides, it can be seen that in the composites, a broad peak at 726.6 cm^−1^ corresponding to heptazine ring breathing mode and stretching vibration modes of C=N heterocycles in g-C_3_N_4_ can be observed^39^, while this peak appears at 708.7 cm^−1^ for CN-500. A peak shift of about 17.9 cm^−1^ may come from the denser packing of the g-C_3_N_4_ layers in the composites as observed from the XRD data. These above XRD, FTIR, and Raman data further supports the success of the method in preparing the composites containing two components of g-C_3_N_4_ and MoS_2_.

Thermal properties of the composites were also studied by thermo-gravimetric analysis (TGA) (Fig. 1d). Accordingly, for all the samples, there are clearly two steps of losing weight. The first step, from room temperature to around 345 °C, may be attributed to the evaporation of physically adsorbed water. The second step corresponding to main weight loss occurs above 345 °C which could be ascribed to the oxidation of MoS_2_ to MoO_3_^26^. This is also ascribed to the decomposition of g-C_3_N_4_ in the composites which happens at higher temperature (> 500 °C) in case of pure g-C_3_N_4_^26^. The reduction of decomposition temperature of g-C_3_N_4_ component in composites indicates the crystallization disturbance of MoS_2_ towards interlayer stacking motifs of g-C_3_N_4_ or the catalyzing effect of MoS_2_ over thermal decomposition of g-C_3_N_4_^16,25,40^. Assuming that the final product after 600 °C is pure MoO_3_, MoS_2_ contents in the samples can be estimated to be 50.7, 55.8, 68.4 and 70.0% for MCN-450, MCN-500, MCN-550, MCN-600, respectively. These results are consistent with the reduction in composition of g-C_3_N_4_ via the increase of treatment temperature observed in XRD and FTIR data.

**Figure 2:**
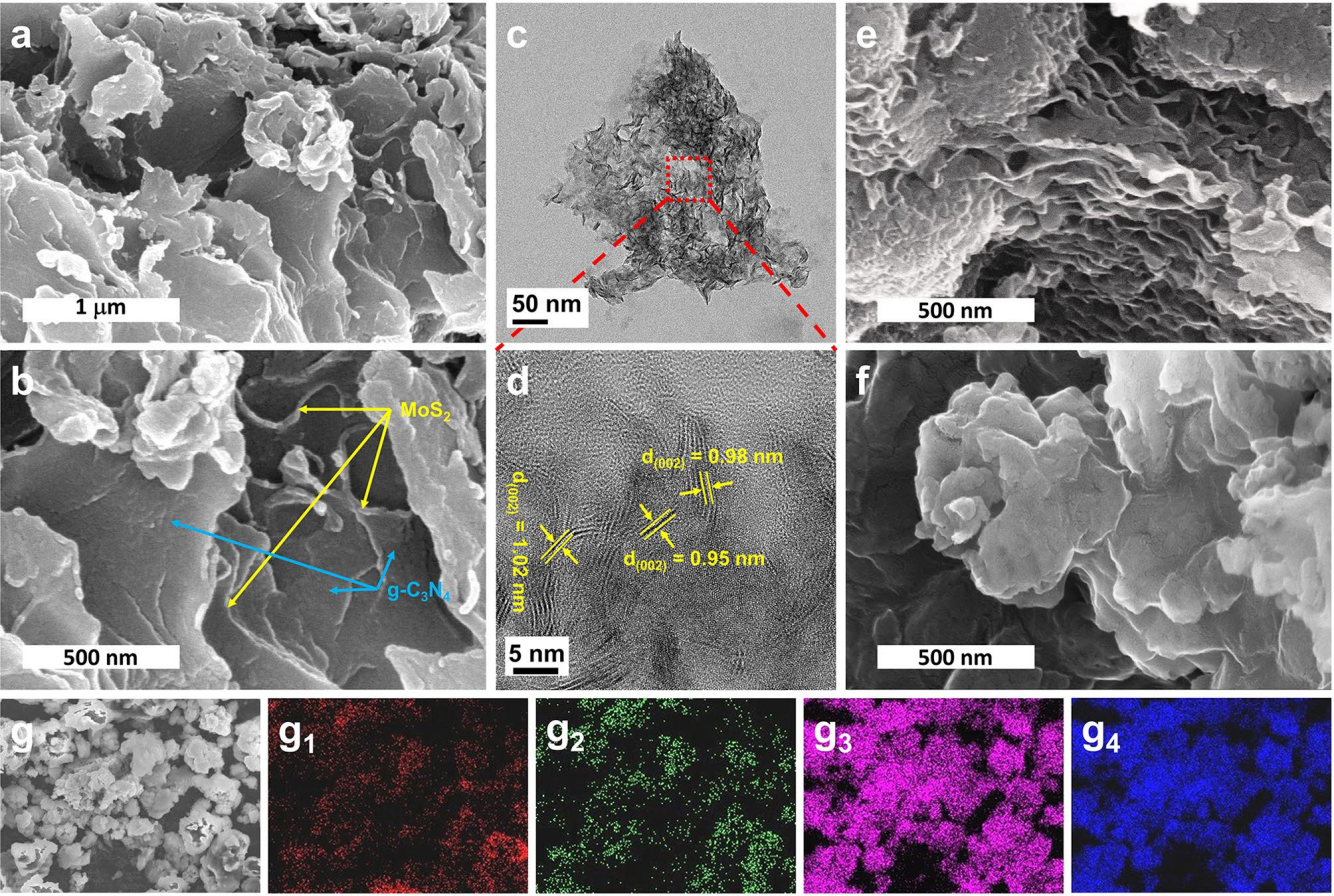
(**a**, **b**) FE-SEM; and (**c**, **d**) HR-TEM images of MCN-500; FE-SEM images of (**e**) MCN-600; and (**f**) CN-500; EDS mapping images of (**g**) mapping area, (**g**_**1**_) carbon, (**g**_**2**_) nitrogen, (**g**_**3**_) sulfur, and (**g**_**4**_) molybdenum.

In Fig. 2, the morphology and elemental composition of representatives were investigated using field emission scanning electron microscopy (FE-SEM), high resolution transmission electron microscopy (HR-TEM), along with energy dispersive X-ray spectroscopy (EDS) mapping. As shown in Fig. 2a,b, the MCN500 was observed as alternately stacking construction in which MoS_2_ nanosheets are identified by their curved edges and g-C_3_N_4_ performs as discontinuous covering layer. The TEM image of MCN-500 in Fig. 2c illustrates a well-dispersion of MoS_2_ in g-C_3_N_4_. At higher magnification, HR-TEM image of this sample (Fig. 2d) indicates that the nanosheets of MoS_2_ present in few-layer bunches with an interlayered spacing of (100) plane around 1 nm which is consistent with observations in XRD and Raman results. This could be explained due to the exposure of gas during decomposition of thiourea leading to higher exfoliation degree of MoS_2_. However, at higher temperatures, MoS_2_ nanosheets tend to reconstruct into denser structure as shown in Fig. 2e. This could be ascribed to mostly removal of g-C_3_N_4_ in MCN600 composite leading to collapse of MoS_2_ nanosheet. This observation could be confirmed by specific surface area determined using Brunauer–Emmett–Teller (BET) measurement as shown in Supplementary Fig. 2. Therein, the specific surface area of MCN-500 is 48.78 m^2^ g^−1^, much higher than that of pure g-C_3_N_4_ (14.44 m^2^ g^−1^). The reduction in specific surface area of MCN-600 (29.35 m^2^ g^−1^) compared to MCN-500 could demonstrate the collapse of MoS_2_ nanosheets due to the decomposition of g-C_3_N_4_ framework. In addition, the elemental mapping images shown in Fig. 2g_1–4_ indicate a homogeneous distribution of composed elements such as carbon, nitrogen, molybdenum, and sulfur.

**Figure 3:**
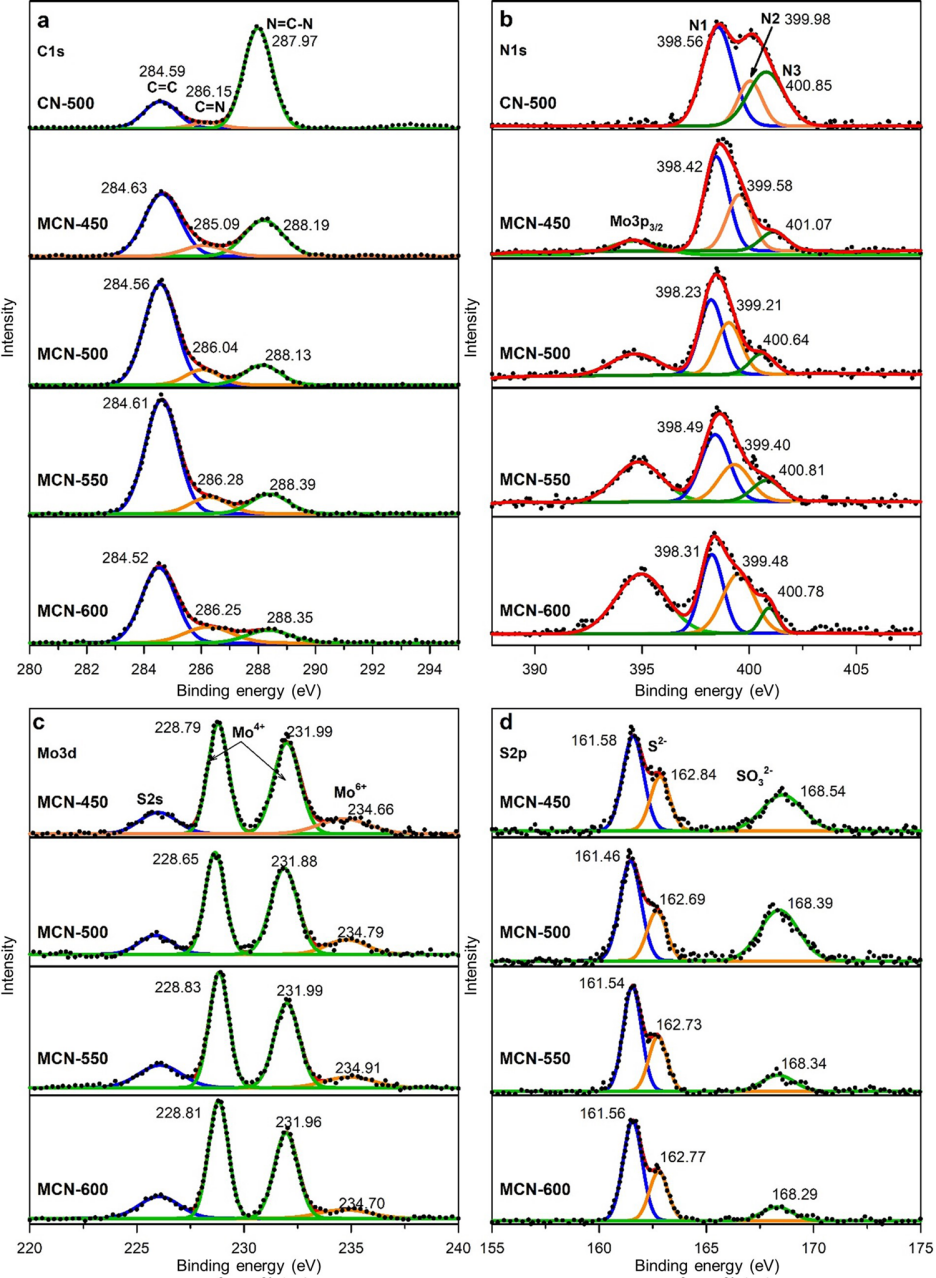
(**a**) C1s; (**b**) N1s XPS spectra of CN-500 and MCN-T; (**c**) Mo3d; and (**d**) S2p XPS spectra of composites MCN-T (T = 450, 500, 550, and 600).

The chemical composition and elemental state on the surface of the as-prepared samples MCN-T were analyzed using X-ray photoelectron spectroscopy (XPS) measurement and the obtained results were present in Fig. 3. In case of g-C_3_N_4_, the C1s spectrum in Fig. 3a comprises of three constituents including graphitic C=C bonding at 284.6 eV, C=N species located at ~ 286.2 eV and aromatic sp^2^-C in N=C–N at ~ 287.9 eV^41^. The C1s of composites perform similar components but composition of these species significantly changes in presence of MoS_2_. It can be observed that, there is an apparent increase in C=C content. Furthermore, as elevating synthesis temperature, the proportion of N-bonded C-species reduces considerably which is consistent with the fact of N-gas release during the condensation of g-C_3_N_4_. In fact, the localized nature of π-electrons in C=N conjugation system^42,43^ is assigned for the limitation in transport of electron then reduce electronic conductivity of pristine g-C_3_N_4_. Therefore, the additional C=C bonds with non-localized π-electrons could expect to improve the charge transfer process in composites. The deficiency of N-content could also be observed from the N1s spectra as shown in Fig. 3b. Accordingly, the N1s spectra of g-C_3_N_4_ could be deconvoluted into three characteristic peaks including the N1 located at 398.6 eV corresponding to the pyridinic sp^2^-hybridized N in C=N–C groups of aromatics rings, the N2 component sited at 399.9 eV ascribed to the pyrrolic N, and the N3 peak centered at around 400.8 eV related to the graphitic ternary nitrogen or bridging N-(C)_3_ in connecting bridges between tri-s-triazine units in structure of g-C_3_N_4_^34,44^. The peak at a lower binding energy of 395 eV ascribed as the signal of Mo3p_3/2_^45^. It is observable that, in addition of MoS_2_, there is a significant decrease in content of N-related groups according to increase of synthesis temperature which confirms the partial N-degradation of g-C_3_N_4_ at high temperature. In addition, compared to pure g-C_3_N_4_, the content of ternary N and pyridinic N in composites apparently decreases while pyrrolic N portion increase as elevation of treatment temperature. There is a fact that the sp^3^ orbitals in bridging N-(C)_3_ are well-known to be tilted out of π-conjugation plane of tri-s-triazine units. This results in a confinement effect to limit π-electrons to travel throughout the whole π-conjugation of g-C_3_N_4_. In other words, the bridging N is the origin of low electrical conductivity of pristine g-C_3_N_4_. Therefore, the reduction of N-content, especially bridging N, is expected to provide more favorable transport for charge carriers in composites. Furthermore, the reduction in N content or the transformation of g-C_3_N_4_ toward N-doped graphene could be confirmed using Raman spectra in range of 1000–1800 cm^−1^ as shown in Supplementary Fig. 3. Accordingly, from MCN-500, the peaks at ~ 1354 cm^−1^ (D band) and ~ 1605 cm^−1^ (G band) obviously emerge as well-known characterization of carbon materials. It is commonly reported the temperature for transformation of g-C_3_N_4_ to graphene-like carbon materials is 730 °C^46^. However, in the case with presence of a suitable catalyst the process could be accelerate at lower temperature^47,48^. Herein, the MoS_2_ could play as a catalyst to promote the transformation of g-C_3_N_4_ into graphitized carbon. The I_D_/I_G_ of MCN-500, -550, and -600 are 0.91, 1.11, and 1.14, respectively, indicating the high disorder degree of graphitic carbon structure and consistent to the reduction in content of graphitic N as well as the increase of defect pyrrolic N. The reduction in N-content and the direct graphitization of g-C_3_N_4_ were expected to enhance the charge transport in composites.

Furthermore, Fig. 3c showed the deconvoluted Mo_3_d spectra of composites containing four peaks. The two high intensity peaks located in the middle region could be ascribed to the Mo_3_d_3/2_ (~ 231.90 eV) and Mo_3_d_5/2_ (~ 228.80 eV) corresponding to main oxidation state of Mo^4+^^16,49^. The low and broaden peak located at around 234.70 eV corresponding to the residual Mo^6+^ which is not reduced but its contribution decrease following the increase of temperature determining that higher treatment temperature is more favorable for complete growth of MoS_2_. The remaining peak is ascribed to the presence of S^2−^, which is characteristic of MoS_2_^50^. The XPS spectra of S2p (Fig. 3d), could be decomposed into three peaks at 168.5, 162.8 and 161.6 eV corresponding to S^4+^ species in SO_3_^2−^ groups, S2p_1/2_ and S2p_3/2_ of MoS_2_, respectively^51^. The peak at 168.5 eV is intense for MCN-450 and MCN-500, and almost disappears in MCN-550 and MCN-600. This may be explained by the fact that SO_3_^2−^ groups as Na_2_SO_3_ forms at 450–500 °C but decompose from 550 °C^19^. From the above analysis, it is clear that composites of MoS_2_/g-C_3_N_4_ can be formed at 450 °C from the precursors of Na_2_MoO_4_ and thiourea. However, at this temperature, polycondensation of g-C_3_N_4_ is incomplete, which is evidenced by the presence of a significant content of C=N–C group. The continuous polycondensation and partly decomposition of g-C_3_N_4_ at higher temperature may occur.

### Photocatalytic activity

**Figure 4:**
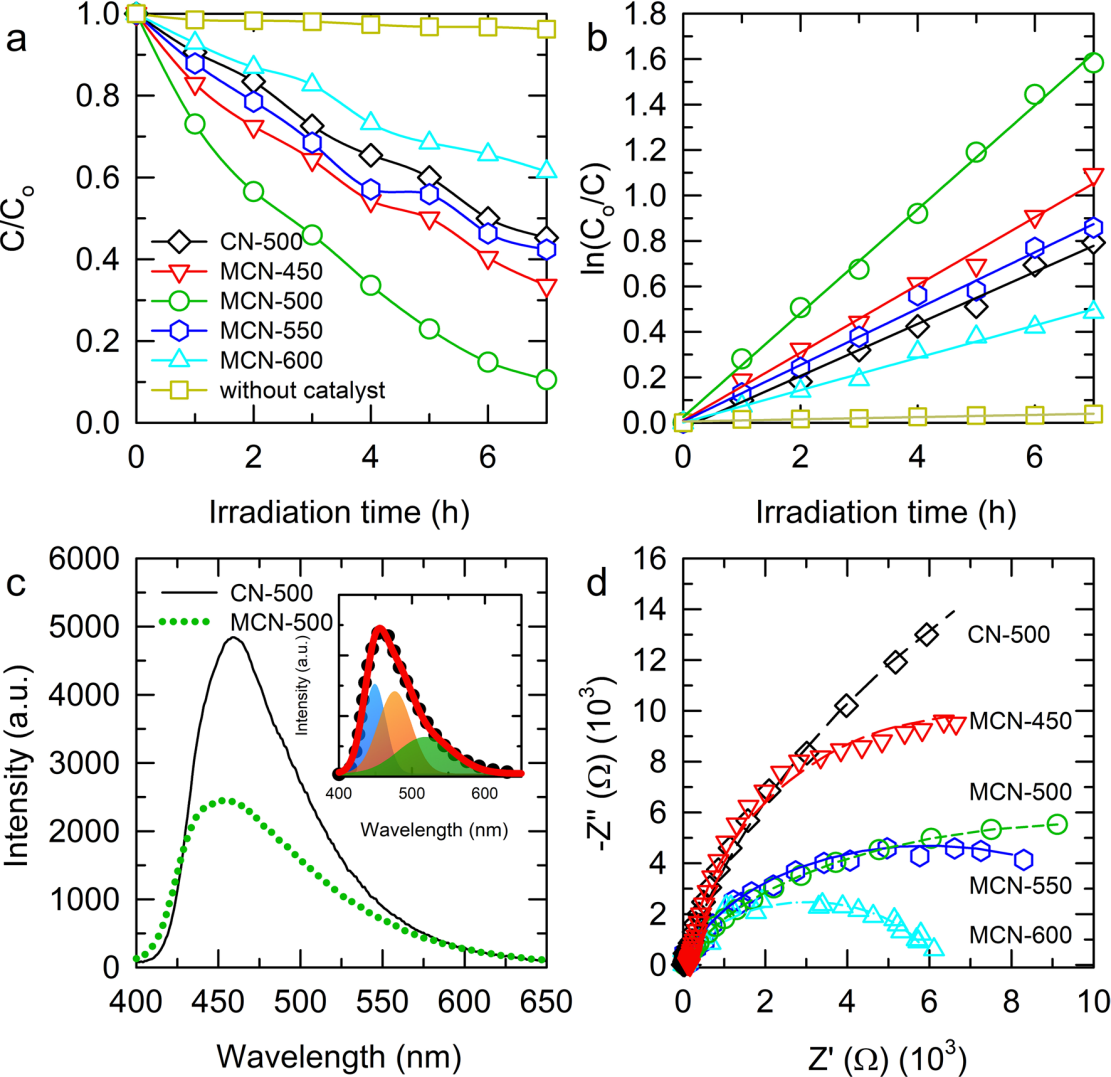
(**a**) Photocatalytic activity on RhB degradation; and (**b**) kinetic analysis of CN-500 and MCN-T; Photoluminescence (PL) spectra of CN-500 and MCN-500; and Nyquist plots of electrochemical impedance spectroscopy (EIS) of CN-500 and MCN-T (T = 450, 500, 550, and 600).

The photocatalytic performance of as-synthesized samples was investigated using a 30 mg L^−1^ solution of RhB as modeling organic pollutant. The plots on variation of RhB concentration by reaction time of all the composites as well as pure g-C_3_N_4_ (CN-500) were present in Fig. 4a. Accordingly, among the experimental samples, MCN-500 exhibits the highest degradation efficiency of 90% while the pure g-C_3_N_4_ conducts degradation for 55% of initial RhB after 6-h of irradiation. Normally, in order to evaluate the kinetic of photocatalytic progress, the Langmuir–Hinshelwood model has been applied^24,27,52^. Figure 4b showed the linear relationship of ln(C_o_/C) vs. irradiation time, in which the well-fitting indicates that the photodegradation of RhB on MCN-T catalysts undergoes as a pseudo-first-order reaction according to the equation: ln(C_o_/C) = k·t, where C (mg L^−1^) is the equilibrium concentration of RhB, C_o_ (mg L^−1^) is the initial concentration of RhB before irradiation, t (h) is the reaction time, and k (h^−1^) is the reaction rate constant. Accordingly, the rate constants of MCN-450, MCN-500, MCN-550, MCN-600 and CN-500 are 0.14901, 0.22851, 0.12400, 0.07125, and 0.11450 h^−1^, respectively. This means that the MCN-450, -500, and -550 composites showed the higher photocatalytic performance compared to that of the pure g-C_3_N_4_. A control experiment in the absence of catalyst show an ignored conversion of RhB which indicates that the decomposition of RhB is not a thermal- or photo-degradation. The enhanced photocatalytic activity of composites MCN-450, -500, and -550 could be ascribed to the synergistic effect between two components of MoS_2_ and g-C_3_N_4_. The gas released from formation and partial decomposition of g-C_3_N_4_ caused a stronger exfoliation of MoS_2_ in composites leading to more active sites exposure which favorable for hetero-interfacial reaction like photocatalytic process. In contrast, the presence of MoS_2_ with narrow band gap accelerates the light harvesting properties of g-C_3_N_4_. As shown in Supplementary Fig. 4a, the UV–Vis DRS absorption data of g-C_3_N_4_ exhibits an absorption band edge at around 440 nm. Meanwhile, the addition of MoS_2_ counterpart evidently extents absorption band of composites deeply into visible region which indicates that MoS_2_ could act as a photo-sensitizer to improve light harvesting properties of g-C_3_N_4_. The photocatalytic performance of MoS_2_/g-C_3_N_4_ composites obtained from our one-pot synthesis was compared to the other reported works as summarized in Supplementary Table 1.

In addition, as discussed in the introduction, it is expected to form a heterojunction between two semiconductors through which the photo-induced electrons and holes can transfer to enhance the charge carriers’ separation. To clarify this process, experiments including photoluminescence (PL), electrochemical impedance spectroscopy (EIS), Mott–Schottky measurements were conducted. The PL results of g-C_3_N_4_ and representative MCN-500 at excitation of 325 nm were present in Fig. 4c. Both of these samples exhibit a broad emission which could be decomposed into three components as shown in the inset of Fig. 4c. According to previous studies on PL properties of g-C_3_N_4_, its electronic band structure is constructed from band of σ(sp^3^C–N) bonds, π(sp^2^C–N) bonds, and the lone pair (LP) electrons of bridging N^53–55^. Therefore, three components in PL emission of g-C_3_N_4_ at 448.6, 475.9, and 517.9 nm corresponding to energy gaps of 2.76, 2.60, and 2.39 eV could be assigned to π* → π, σ* → LP, and π* → LP transition pathways^11,56^. In pure g-C_3_N_4_, the recombination process could occur between LP donor of N-atoms to aromatic acceptor^57,58^. However, in composite sample, the PL intensity is significantly suppressed which indicates the recombination of photo-induced charge carriers in composites is prevented or, in other words, the electron–hole separation lifetime is expanded. One of the reasons for the reduction in recombination rate could be assigned to the enhancement of charge transfer process. As mentioned in XPS discussion, the blocking effect of non-conjugated orbitals of bridging N could lead to the confinement of π-electrons in local tri-s-triazine units. With the presence of MoS_2_, a low N-content structure for g-C_3_N_4_ could perform better charge transfer as demonstrated in EIS results. As shown in Fig. 4d, the Nyquist plots of pure g-C_3_N_4_ and all the synthesized composites electrodes are composed of uncompleted semicircular arc. Generally, this semicircular diameter determines how fast and effective the transport and separation of photo-generated electron–hole pairs are on the surface of electrodes. The larger semicircle, the higher charge transfer resistance, and the less effective of charge carriers’ separation^59–61^. According to Fig. 4d, the presence of MoS_2_ in composites MCN-500, -550, and -600 causes a critical reduction in charge transfer resistance. The charge transport is significantly improved via increase of synthesis temperature which could be attributed to the reduction of N-content and delocalization of π-electron system of g-C_3_N_4_. This graphitization toward carbon materials of g-C_3_N_4_ as demonstration in XPS and Raman spectra could be a reasonable explanation for the improvement in charge transport via the elevation of temperature. Nevertheless, the optimization in photocatalytic activity of MCN-500 could be explained due to the fact that, at higher temperature, the lower content of g-C_3_N_4_ leads to the restacking of MoS_2_ nanosheets as observed in Fig. 2e and BET results. This re-aggregation of MoS_2_ nanosheets turns it to charge recombination center leading to poor photocatalytic performance.

**Figure 5:**
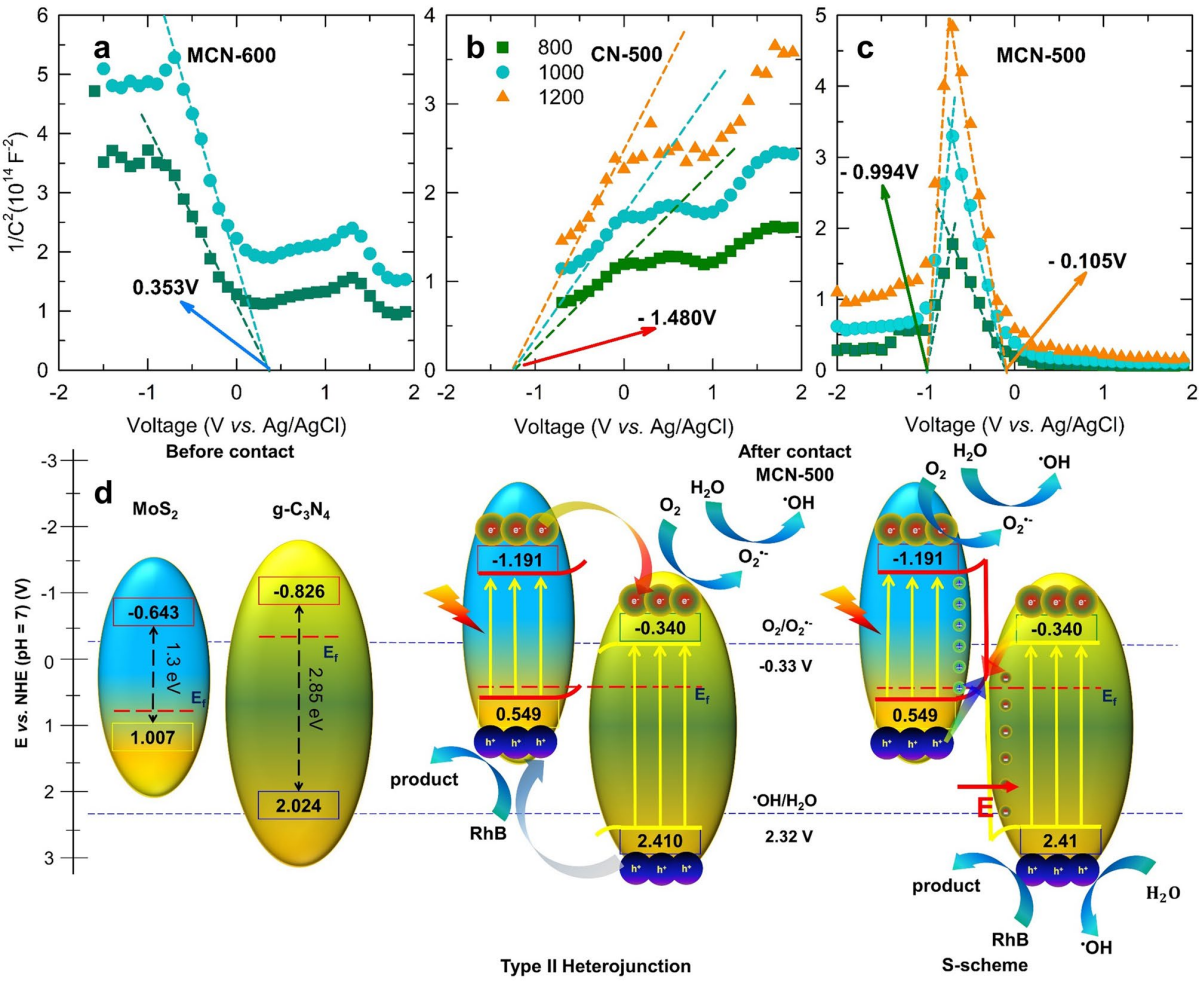
Mott–Schottky plots of (**a**) MCN-600, (**b**) CN-500; and (**c**) MCN-500; (**d**) schematic diagram of band alignment of heterojunction and S-scheme charge transfer on interface of MoS_2_ and g-C_3_N_4_.

To determine the band edges position and further information in electronic structure of obtained samples, the Mott–Schottky measurement was conducted on three samples such as MCN-600, CN-500 and MCN-500. The obtained data was collected from the impedance spectroscopy carried out in the voltage range from − 2.0 to 2.0 V with step of 0.1 V at three different frequency of 800, 1000 and 1200 Hz. As shown in Fig. 5a,b, the Mott–Schottky plot of MCN-600 shows a negative slope which is characterized for p-type semiconductor corresponding to MoS_2_ with *x*-axis intercept of 0.353 V (vs*.* SSCE) while that of CN-500 shows a positive slope corresponding to typical n-type with flat band voltage as − 1.48 V (vs. SSCE). As the feature of p-type semiconductor, the collected flat band potential of MoS_2_ could be ascribed to the valence band maximum (VBM) while the correlative value of CN-500 was indicated as conduction band minimum (CBM) due to n-type characterization^25,62^. Accordingly, via the Nernst equation, the potential vs. reversible hydrogen electrode (RHE) or normal hydrogen electrode (NHE) at pH = 7, could be converted from obtained data:

1 $$E_{RHE} = E_{electrode} + E_{SSCE}^{^\circ } + 0.059pH$$

in which, E_RHE_ corresponding to potential vs. RHE, E_electrode_ indicating for the collected value of working electrode, and $E_{SSCE}^{^\circ }$ ascribed to the standard potential of SSCE as 0.241 V. As the results, the position of VBM edge for MoS_2_ in MCN-600 and CBM edge for g-C_3_N_4_ were calculated as 1.007 V and − 0.826 V, vs. RHE, respectively. Furthermore, the Mott-Schottky plot of MCN-500 composite (Fig. 5c) exhibits a reverse V-shape with two distinguish regions which is characterized for the p–n junction formation between MoS_2_ and g-C_3_N_4_^25,62^. Accordingly, for MCN-500, the VBM of MoS_2_ located at − 0.105 V (vs. SSCE) or 0.549 V (vs. RHE), while the CBM of g-C_3_N_4_ sited at − 0.994 V (vs. SSCE) or − 0.340 V (vs. RHE). In addition, using Kubelka–Munk equation for DRS data, the optical band gap of g-C_3_N_4_ and MoS_2_ could be derived as shown in Supplementary Fig. 4b–d. Therefore, the remaining CBM and VBM of MoS_2_ and g-C_3_N_4_ and these components in MCN-500 could be calculated as illustrated in Fig. 5d. By the formation of p–n contacting region in composite, the band position of both MoS_2_ and g-C_3_N_4_ was shifted to ensure Fermi levels reach to new balanced state which is resulted in the final electronic structure of p-n heterojunction.

In theoretical concept of synergic effect in bi-semiconductor system to enhance the separation of photo-generated electron–hole pairs, the type II heterojunction and S-scheme diagram is the most popular models. In details, in the case of type II heterojunction model, after being excited by photons with sufficient energy, electrons could be activated to jump from VB to CB. Thereby, due to the more positive value of VB in g-C_3_N_4_, electrons could transfer from CB of MoS_2_ to CB of g-C_3_N_4_ while holes follow the opposite trends in VB. As the result, the photo-induced electrons and holes were separated and last for longer lifetime. However, in terms of dynamic, the repulsion of the same charged carriers (electrons on CB and holes on VB of MoS_2_ and g-C_3_N_4_) could prohibit the transfer of them. Similarly, the attraction of electrons and hole in individual semiconductor also inhibits the transport of electrons on CB and holes on VB. Furthermore, the charge transfer following type II heterojunction leading to accumulation of the main oxidative holes on VB of MoS_2_ and the main reductive electrons on CB of g-C_3_N_4_ which both are weaker driving force. Meanwhile, in step scheme (S-scheme) model, as report by density functional theory calculation in the previous publications^63–65^, the work function of g-C_3_N_4_ (4.67 eV) is much lower than that of MoS_2_ (5.69 eV) leading to electron transfer from g-C_3_N_4_ to MoS_2_ at their heterojunction interface. This induces an electron depletion region on MoS_2_ and an electron accumulation layer on g-C_3_N_4_ at their interface. By other words, the surface of MoS_2_ turns into positive charge while the negatively charged state is observed in contact region of g-C_3_N_4_. The polarization of charge at interface of these components could raise an internal electric field (IEF) directing from MoS_2_ to g-C_3_N_4_. This IEF could promote the transport of electrons in opposite direction, from g-C_3_N_4_ to MoS_2_. In addition, the alignment of Fermi levels at the interface leads to upward and downward shift of band positions of MoS_2_ and g-C_3_N_4_, respectively. This band bending accelerates the photo-induced charged carriers on CB of g-C_3_N_4_ and VB of MoS_2_ to transport toward the interface and recombine there. Furthermore, this recombination is also driven by the electrostatic attraction of positive charge of holes on VB of MoS_2_ and negative charge of electron on CB of g-C_3_N_4_. Therefore, all the driving forces including the formation of IEF, the band bending, and the electrostatic attraction, expedite the recombination of electrons on CB of g-C_3_N_4_ and holes on VB of MoS_2_ which keeps longer separation of electrons on CB of MoS_2_ and holes on VB of g-C_3_N_4_ for photocatalytic redox reactions. All the photo-induced charge carriers transport following S-scheme was illustrated in Fig. 5d. ^63,66,67^. ***This charge transmission could be demonstrated by the surface photovoltage profile as shown in Supplementary Fig. 5. Accordingly, the photo-response signal observed in MCN-500 is much higher than those of pure g-C_3_N_4_ and MCN-600 which indicates better charge transport of MCN-500. In addition, the preserved photo-generated electrons and holes in S-scheme located at higher CB and VB than those in type II heterojunction which leads to stronger driving force for redox reactions in photocatalytic activity. Theoretically, the oxygen reduction of electron on CB leads to formation of O_2_^·−^ species while water oxidation of hole on VB fabricates the ^·^OH radicals. The VB position of MoS_2_ in MCN-500, however, is more negative than redox potential of ^·^OH/H_2_O (= 2.32 V vs. NHE at pH = 7)^1,2^, so it is impossible to form the ^·^OH radicals directly from type II heterojunction model. Therefore, the production of ^·^OH radicals in type II heterojunction mechanism is only based on the further multi-electron redox step from O_2_^·−^. All the aforementioned processes are summarized in following equation^1,2^:

2 $${\text{e}}^{ - } + {\text{O}}_{2} \to {\text{O}}_{2}^{ \cdot - }$$

3 $${\text{O}}_{2}^{ \cdot - } + {\text{H}}^{ + } \to {}^{ \cdot }{\text{OOH}}$$

4 $${}^{ \cdot }{\text{OOH}} + {\text{H}}^{ + } + {\text{e}}^{ - } \to {\text{H}}_{2} {\text{O}}_{2}$$

5 $${\text{H}}_{2} {\text{O}}_{2} + {\text{e}}^{ - } \;{}^{ \cdot }{\text{OH}} + {\text{OH}}^{ - }$$

Nevertheless, both CB of MoS_2_ and g-C_3_N_4_ are more negative than reduction potential of O_2_/O_2_^·−^ (= − 0.33 V, vs. NHE at pH = 7)^1,2,4^, which implies that direct reduction of dissolved oxygen to form ${\text{O}}_{2}^{ \cdot - }$ species is possible in both cases. However, the higher CB position of MoS_2_ is more favorable for direct oxygen reduction. Based on the quenching results as shown in Supplementary Fig. 6, in the presence of TB, the RhB degradation efficiency is the lowest which indicates that ^·^OH radicals are dominant contribution in photocatalytic activity of MCN-500. Meanwhile, when adding BQ, the efficiency decreases insignificantly compared to case of TB which demonstrates that the O_2_^·−^ anion is not the main active species in photocatalytic activity of MCN-500. In other words, the mechanism via S-scheme model with direct formation of ^·^OH is more suitable to explain these experiments.

## Discussions

In this work, the MoS_2_/g-C_3_N_4_ composites were synthesized by direct calcination of sodium molybdate and thiourea precursors at different temperatures from 450 to 600 °C. The applied temperature not only alters the composition of final components but also controls the exfoliation degree of MoS_2_ via formation and partial decomposition of g-C_3_N_4_. The presence of MoS_2_ extends the light harvesting range of composite toward near infrared region while the reduction in N-content and partial transformation to graphitic carbon of g-C_3_N_4_ provide more delocalized π-electrons conjugation for improvement in the charge transfer process. The photocatalytic activity of composites is investigated on RhB degradation in which MCN-500 performs the highest conversion efficiency. This enhancement is attributed to more effective photo-generated electron–hole pairs separation following an S-scheme charge transfer pathway.

## Methods

### Preparation of materials

All the chemicals were purchased from Sigma-Aldrich and used without further purification. MoS_2_/g-C_3_N_4_ composites were synthesized by calcining the mixture of sodium molybdate dehydrate, Na_2_MoO_4_^.^2H_2_O (99% purity) and thiourea, CH_4_N_2_S (99% purity) with a mass ratio of 1:3 at various temperatures. In a typical synthesis, a mixture of Na_2_MoO_4_·2H_2_O and thiourea was well-grinded, transferred into a ceramic crucible covered by aluminum foil, and then heated in a tube furnace at different temperatures of T (T = 450 °C, 500 °C, 550 °C, and 600 °C) for 1 h with heating rate of 10 °C/min under N_2_ gas flow. The as-prepared samples were washed with water and ethanol to eliminate the sodium salts and residual organic side products, which were in denoted as MCN-T. For comparison, pure g-C_3_N_4_ was prepared by calcining thiourea at 500 °C for 1 h under N_2_ gas flow, and then washing with water and ethanol as mentioned above for the preparation of MCN-T, which was referred as CN-500.

### Material characterization

XRD analysis was carried using a D8 Advanced Bruker anode X-ray Diffractometer with Cu Kα (λ = 1.5406 Å) radiation. The morphology of the synthesized samples was characterized by FE-SEM while the elemental mapping images were obtained using EDS which both were investigated on JEOL JSM-600F. HR-TEM images were obtained using a JEOL JEM-2100F. FT-IR spectra of the samples were recorded using an IR Prestige-21 spectrophotometer (Shimadzu). Raman spectra were conducted on T64000 Raman using a 647.1 nm laser as excitation source and detector CCD was cooled by liquid nitrogen. The thermogravimetric-differential scanning calorimetry analysis was carried out on the SETRAM LABSYS TG system under air flow with heating rate of 10 °C/min. Specific surface area of as-prepared samples was determined using Brunauer–Emmett–Teller (BET) on TriStar 3000. XPS was conducted by Theta Probe AR-XPS System (Thermo Fisher Scientific). PL spectra were investigated using the Perkin-Elmer LS-55 Fluorescence Spectrometer. The Mott–Schottky results were recorded in a voltage range of − 2.0 to 2.0 V at different frequencies of 800, 1000, and 1200 Hz while the EIS was carried out under applied AC voltage of 10 mV in frequency from 100 mHz to 10 kHz which were both conducted on AutoLab M101. These measurements were accomplished in three-electrode system using sodium saturated calomel electrode (SSCE) and Pt electrode as reference and counter while the aqueous solution of 0.2 M Na_2_SO_4_ (pH = 7) was applied as electrolyte. A slurry of selected samples in ethanol was cast on fluorine doped tin oxides (FTO) glass (1 × 1 cm) and dried on a hotplate for fabricating working electrodes. Steady-state surface photovoltage was recorded using self-assembled surface photovoltaic technique based on a lock-in amplifier (SR830-DSP) with a Xenon lamp as light source.

### Photocatalytic experiments

Photocatalytic activity of the samples was evaluated by degrading rhodamine B (RhB) in aqueous solution. Into 80 mL of the 30 mg/L RhB solution, 0.05 g of the sample was dispersed under continuously stirring and then the solution was kept in dark for 2 h to achieve adsorption–desorption equilibrium before irradiated by a 100 W lamp with a filter cutting off UV rays (200–400 nm). The degradation of RhB was monitored by taking the suspension at the irradiation time intervals of 1 h. The concentration of RhB in the collected solution was determined by measuring the absorbance at 553 nm after removing the catalyst by centrifuge. The quenching experiments were conducted using benzoquinone (BQ), tert-butanol (TB), sodium salt of ethylenediaminetetraacetic acid (EDTA-Na), and dimethyl sulfoxide (DMSO) as quenchers for superoxide anion (O_2_^·−^), hydroxyl (^·^OH) radicals, photo-generated holes, and electrons, respectively.

## Supplementary Information

Supplementary Information.

## Data Availability

The data that support the findings within this paper are available from the corresponding author on request.

## References

[1] Ibhadon AO, Fitzpatrick P (2013). Heterogeneous photocatalysis: Recent advances and applications. Catalysts.

[2] Li J, Wu N (2015). Semiconductor-based photocatalysts and photoelectrochemical cells for solar fuel generation: A review. Catal. Sci. Technol..

[3] Al-Hamdi AM, Rinner U, Sillanpää M (2017). Tin dioxide as a photocatalyst for water treatment: A review. Process Saf. Environ. Protect..

[4] Gupta SM, Tripathi M (2011). A review of TiO_2_ nanoparticles. Chin. Sci. Bull..

[5] Wang X (2009). A metal-free polymeric photocatalyst for hydrogen production from water under visible light. Nat. Mater..

[6] Zhang J, Guo F, Wang X (2013). An optimized and general synthetic strategy for fabrication of polymeric carbon nitride nanoarchitectures. Adv. Funct. Mater..

[7] Yan S, Li Z, Zou Z (2010). Photodegradation of rhodamine B and methyl orange over boron-doped g-C_3_N_4_ under visible light irradiation. Langmuir.

[8] Jun YS (2013). From melamine-cyanuric acid supramolecular aggregates to carbon nitride hollow spheres. Adv. Funct. Mater..

[9] Zhang Y, Liu J, Wu G, Chen W (2012). Porous graphitic carbon nitride synthesized via direct polymerization of urea for efficient sunlight-driven photocatalytic hydrogen production. Nanoscale.

[10] Zhang J (2011). Sulfur-mediated synthesis of carbon nitride: Band-gap engineering and improved functions for photocatalysis. Energy Environ. Sci..

[11] Tran DA (2021). One-step synthesis of oxygen doped g-C_3_N_4_ for enhanced visible-light photodegradation of Rhodamine B. J. Phys. Chem. Solids.

[12] Yang S (2013). Exfoliated graphitic carbon nitride nanosheets as efficient catalysts for hydrogen evolution under visible light. Adv. Mater..

[13] Kappadan, S., Thomas, S. & Kalarikkal, N. Enhanced photocatalytic performance of BaTiO_3_/g-C_3_N_4_ heterojunction for the degradation of organic pollutants. *Chem. Phys. Lett.* **771**, 138513 (2021).

[14] Van KN (2021). A novel preparation of GaN-ZnO/g-C_3_N_4_ photocatalyst for methylene blue degradation. Chem. Phys. Lett..

[15] Yu W, Xu D, Peng T (2015). Enhanced photocatalytic activity of gC_3_N_4_ for selective CO_2_ reduction to CH_3_OH via facile coupling of ZnO: A direct Z-scheme mechanism. J. Mater. Chem. A.

[16] Tran Huu H, Nguyen Thi XD, Van Nguyen K, Kim SJ, Vo V (2019). A facile synthesis of MoS_2_/g-C_3_N_4_ composite as an anode material with improved lithium storage capacity. Materials.

[17] Shokri A, Salami N (2016). Gas sensor based on MoS_2_ monolayer. Sens. Actuators B-Chem..

[18] Sarkar D (2019). Expanding interlayer spacing in MoS_2_ for realizing an advanced supercapacitor. ACS Energy Lett..

[19] Li M (2016). Dual synergetic effects in MoS_2_/pyridine-modified g-C_3_N_4_ composite for highly active and stable photocatalytic hydrogen evolution under visible light. Appl. Catal. B-Environ..

[20] Chen Y, Tian G, Shi Y, Xiao Y, Fu H (2015). Hierarchical MoS_2_/Bi_2_MoO_6_ composites with synergistic effect for enhanced visible photocatalytic activity. Appl. Catal. B-Environ..

[21] Xia J (2015). Microwave-assisted synthesis of few-layered MoS_2_/BiOBr hollow microspheres with superior visible-light-response photocatalytic activity for ciprofloxacin removal. CrystEngComm.

[22] Yan J (2016). Construction of a 2D graphene-like MoS_2_/C_3_N_4_ heterojunction with enhanced visible-light photocatalytic activity and photoelectrochemical activity. Chem. Eur. J..

[23] Wang J, Guan Z, Huang J, Li Q, Yang J (2014). Enhanced photocatalytic mechanism for the hybrid gC_3_N_4_/MoS_2_ nanocomposite. J. Mater. Chem. A.

[24] Lu X (2016). Controllable synthesis of graphitic C_3_N_4_/ultrathin MoS_2_ nanosheet hybrid nanostructures with enhanced photocatalytic performance. Dalton Trans..

[25] Liu Y (2018). 0D (MoS_2_)/2D (g-C_3_N_4_) heterojunctions in Z-scheme for enhanced photocatalytic and electrochemical hydrogen evolution. Appl. Catal. B-Environ..

[26] Liu Y (2018). Flower-like MoS_2_ on graphitic carbon nitride for enhanced photocatalytic and electrochemical hydrogen evolutions. Appl. Catal. B-Environ..

[27] Peng W-C, Li X-Y (2014). Synthesis of MoS_2_/g-C_3_N_4_ as a solar light-responsive photocatalyst for organic degradation. Catal. Commun..

[28] Yuan Y-J (2019). Liquid exfoliation of g-C_3_N_4_ nanosheets to construct 2D–2D MoS_2_/g-C_3_N_4_ photocatalyst for enhanced photocatalytic H_2_ production activity. Appl. Catal. B-Environ..

[29] Zhang X, Zhang R, Niu S, Zheng J, Guo C (2019). Enhanced photo-catalytic performance by effective electron-hole separation for MoS_2_ inlaying in g-C_3_N_4_ hetero-junction. Appl. Sur. Sci..

[30] Shi L, He Z, Liu S (2018). MoS_2_ quantum dots embedded in g-C_3_N_4_ frameworks: A hybrid 0D–2D heterojunction as an efficient visible-light driven photocatalyst. Appl. Sur. Sci..

[31] Xue B, Jiang H-Y, Sun T, Mao F, Wu J-K (2018). One-step synthesis of MoS_2_/g-C_3_N_4_ nanocomposites with highly enhanced photocatalytic activity. Mater. Lett..

[32] Liu Y (2013). Preparation, characterization and photoelectrochemical property of ultrathin MoS_2_ nanosheets via hydrothermal intercalation and exfoliation route. J. Alloy Compd..

[33] Li J (2016). Synthesis of MoS_2_/g-C_3_N_4_ nanosheets as 2D heterojunction photocatalysts with enhanced visible light activity. Appl. Sur. Sci..

[34] Papailias I (2015). Effect of processing temperature on structure and photocatalytic properties of g-C_3_N_4_. Appl. Sur. Sci..

[35] Yin L, Hai X, Chang K, Ichihara F, Ye J (2018). Synergetic exfoliation and lateral size engineering of MoS_2_ for enhanced photocatalytic hydrogen generation. Small.

[36] Chang C, Chan S (1981). Infrared and Raman studies of amorphous MoS3 and poorly crystalline MoS_2_. J. Catal..

[37] Frey GL, Tenne R, Matthews MJ, Dresselhaus M, Dresselhaus G (1999). Raman and resonance Raman investigation of MoS_2_ nanoparticles. Phys. Rev. B.

[38] Gołasa K (2014). Resonant Raman scattering in MoS_2_—From bulk to monolayer. Solid State Commun..

[39] Li J (2012). A facile approach to synthesize novel oxygen-doped g-C_3_N_4_ with superior visible-light photoreactivity. Chem. Commun..

[40] Qu Y, Duan X (2013). Progress, challenge and perspective of heterogeneous photocatalysts. Chem. Soc. Rev..

[41] Zang Y, Li L, Li X, Lin R, Li G (2014). Synergistic collaboration of g-C_3_N_4_/SnO_2_ composites for enhanced visible-light photocatalytic activity. Chem. Eng. J..

[42] Chuang P-K, Wu K-H, Yeh T-F, Teng H (2016). Extending the π-conjugation of g-C_3_N_4_ by incorporating aromatic carbon for photocatalytic H2 evolution from aqueous solution. ACS Sustain. Chem. Eng..

[43] Xia P, Cheng B, Jiang J, Tang H (2019). Localized π-conjugated structure and EPR investigation of g-C_3_N_4_ photocatalyst. Appl. Sur. Sci..

[44] Mo Z (2015). Synthesis of gC_3_N_4_ at different temperatures for superior visible/UV photocatalytic performance and photoelectrochemical sensing of MB solution. RSC Adv..

[45] Li R (2017). Nitrogen doped MoS_2_ nanosheets synthesized via a low-temperature process as electrocatalysts with enhanced activity for hydrogen evolution reaction. J. Power Sources.

[46] Li J (2015). Direct transformation from graphitic C_3_N_4_ to nitrogen-doped graphene: An efficient metal-free electrocatalyst for oxygen reduction reaction. ACS Appl. Mater. Interfaces.

[47] Chen J (2017). Nitrogen-deficient graphitic carbon nitride with enhanced performance for lithium ion battery anodes. ACS Nano.

[48] Tang Y (2020). Templated transformation of g-C_3_N_4_ nanosheets into nitrogen-doped hollow carbon sphere with tunable nitrogen-doping properties for application in Li-ions batteries. Carbon.

[49] Zhao L (2017). One-step synthesis of CdS nanoparticles/MoS_2_ nanosheets heterostructure on porous molybdenum sheet for enhanced photocatalytic H_2_ evolution. Appl. Catal. B-Environ..

[50] Zheng D, Zhang G, Hou Y, Wang X (2016). Layering MoS_2_ on soft hollow g-C_3_N_4_ nanostructures for photocatalytic hydrogen evolution. Appl. Catal. A-General.

[51] Vrubel H, Merki D, Hu X (2012). Hydrogen evolution catalyzed by MoS_3_ and MoS_2_ particles. Energy Environ. Sci..

[52] Shi L, Liang L, Wang F, Liu M, Sun J (2015). Enhanced photocatalytic activity of degrading rhodamine B over MoS_2_/g-C_3_N_4_ photocatalyst under visible light. Energy Environ. Focus.

[53] Zhang Y (2013). Synthesis and luminescence mechanism of multicolor-emitting gC_3_N_4_ nanopowders by low temperature thermal condensation of melamine. Sci. Rep..

[54] Wang B, Cheng Q, Wang L, Zheng K, Ostrikov K (2012). The effect of temperature on the mechanism of photoluminescence from plasma-nucleated, nitrogenated carbon nanotips. Carbon.

[55] Wang B, Cheng Q, Chen Y, Ostrikov K (2011). Room-temperature photoluminescence from nitrogenated carbon nanotips grown by plasma-enhanced hot filament chemical vapor deposition. J. Appl. Phys..

[56] Yuan Y (2015). High-yield synthesis and optical properties of gC_3_N_4_. Nanoscale.

[57] Fan X (2015). Construction of graphitic C_3_N_4_-based intramolecular donor–acceptor conjugated copolymers for photocatalytic hydrogen evolution. ACS Catal..

[58] Jin X (2016). MoS_2_ quantum dot decorated gC_3_N_4_ composite photocatalyst with enhanced hydrogen evolution performance. RSC Adv..

[59] Ansari SA, Cho MH (2016). Highly visible light responsive, narrow band gap TiO_2_ nanoparticles modified by elemental red phosphorus for photocatalysis and photoelectrochemical applications. Sci. Rep..

[60] Ansari SA, Cho MH (2017). Simple and large scale construction of MoS_2_-gC_3_N_4_ heterostructures using mechanochemistry for high performance electrochemical supercapacitor and visible light photocatalytic applications. Sci. Rep..

[61] Ansari SA, Ansari MO, Cho MH (2016). Facile and scale up synthesis of red phosphorus-graphitic carbon nitride heterostructures for energy and environment applications. Sci. Rep..

[62] Ke J (2017). Facile assembly of Bi_2_O_3_/Bi_2_S_3_/MoS_2_ np heterojunction with layered n-Bi_2_O_3_ and p-MoS_2_ for enhanced photocatalytic water oxidation and pollutant degradation. Appl. Catal. B-Environ..

[63] Chen Y (2021). One-step construction of S-scheme heterojunctions of N-doped MoS_2_ and S-doped g-C_3_N_4_ for enhanced photocatalytic hydrogen evolution. Chem. Eng. J..

[64] Zhu B, Zhang J, Jiang C, Cheng B, Yu J (2017). First principle investigation of halogen-doped monolayer g-C_3_N_4_ photocatalyst. Appl. Catal. B-Environ..

[65] Gao B (2019). Density functional theory calculation on two-dimensional MoS_2_/BiOX (X = Cl, Br, I) van der Waals heterostructures for photocatalytic action. Appl. Sur. Sc..

[66] Xu Q, Zhang L, Cheng B, Fan J, Yu J (2020). S-scheme heterojunction photocatalyst. Chem.

[67] He F (2020). 2D/2D/0D TiO2/C3N4/Ti3C2 MXene composite S-scheme photocatalyst with enhanced CO_2_ reduction activity. Appl. Catal. B Environ..

