# Supplementary material for: One-pot synthesis of S-scheme MoS2/g-C3N4 heterojunction as effective visible light photocatalyst
Source: Sci Rep. 2021 Jul 20;11:14787. doi: 10.1038/s41598-021-94129-0 (PMC8292365; doi:10.1038/s41598-021-94129-0)
Supplement: Supplementary file 1 — Supplementary Information. [file 41598_2021_94129_MOESM1_ESM.pdf]

Supplementary Information

One-pot synthesis of S-scheme MoS_2/g-C_3N_4 heterojunction as effective visible light photocatalyst

Ha Tran Huu[1*], My Duyen Nguyen Thi[1], Van Phuc Nguyen[1], Lan Nguyen Thi[1], Thi Thuy Trang Phan,[1] Quoc Dat Hoang[2], Huy Hoang Luc[3], Sung Jin Kim[4], Vien Vo[1*]

[1]Faculty of Natural Sciences, Quy Nhon University, 170 An Duong Vuong, Quy Nhon, Binh Dinh 55000, Vietnam

[2]Vietnam Ministry of Science and Technology, 113 Tran Duy Hung, Cau Giay, Hanoi 10000, Vietnam

[3]Faculty of Physics, Hanoi National University of Education, 100000, Vietnam

[4]Department of Chemistry and Nano Science, Ewha Womans University, Seoul 120-750, Korea

[*]Corresponding author: Ha Tran Huu and Vien Vo (E-mail: tranhuuhaqn1992@gmail.com, vovien@qnu.edu.vn)

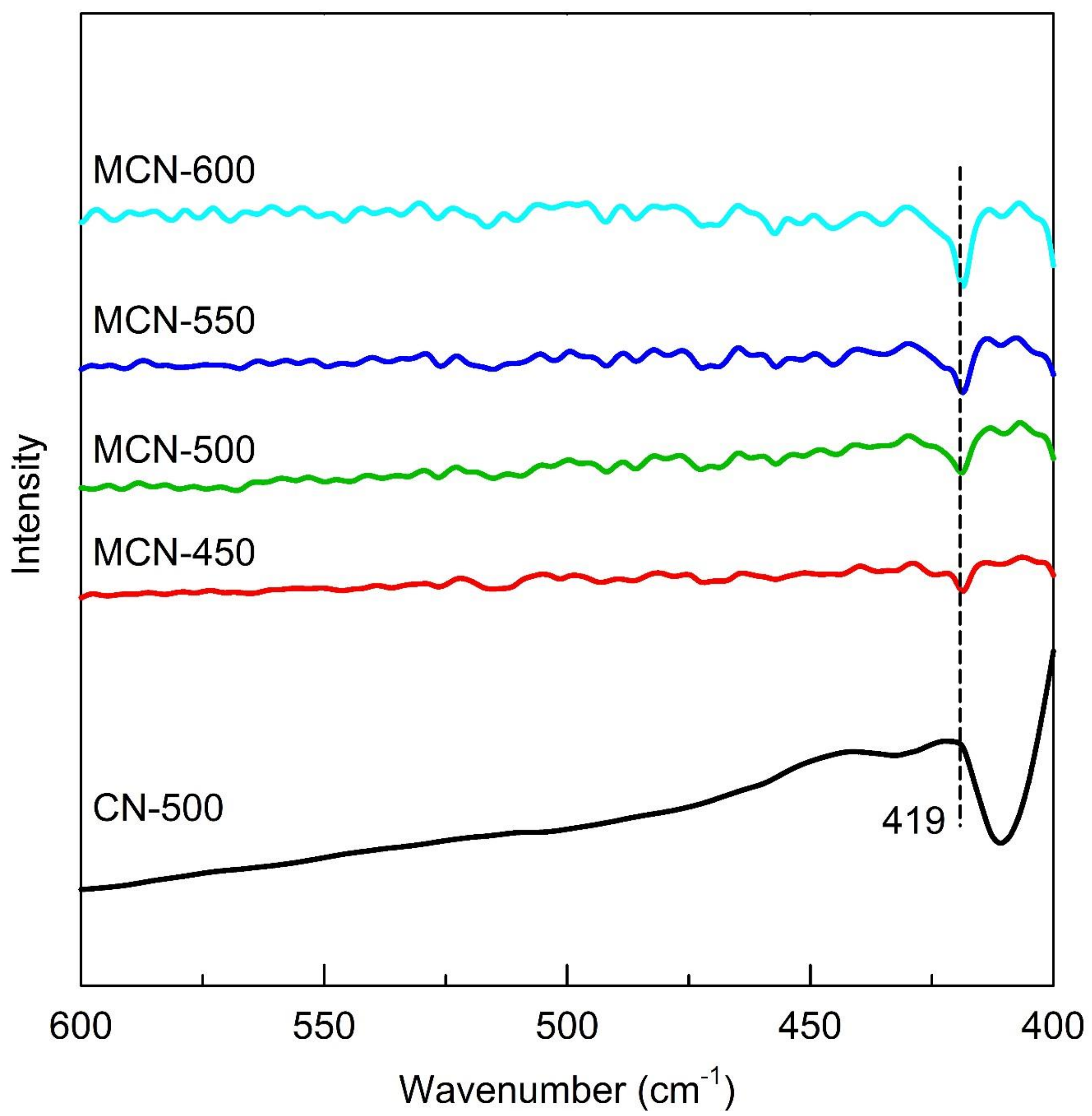

Supplementary Figure 1. Expansion of FT-IR spectra in wavenumber range of 400 – 600 cm^{-1} of CN-500 and MCN-T (T = 450, 500, 550, and 600).

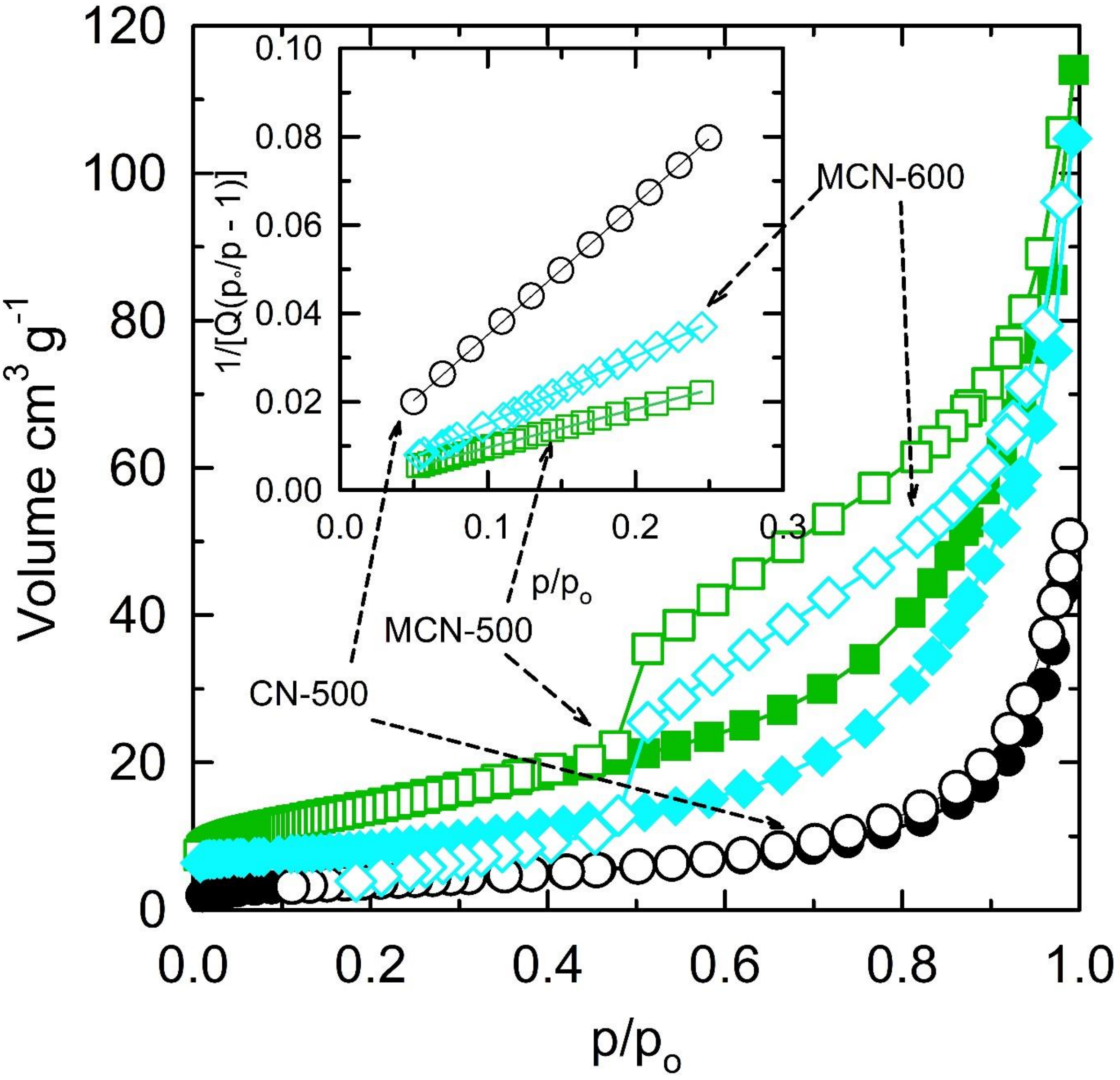

Supplementary Figure 2. N_2 adsorption – desorption curves (inset: linear BET plots) of MCN-500, MCN-600, and CN-500.

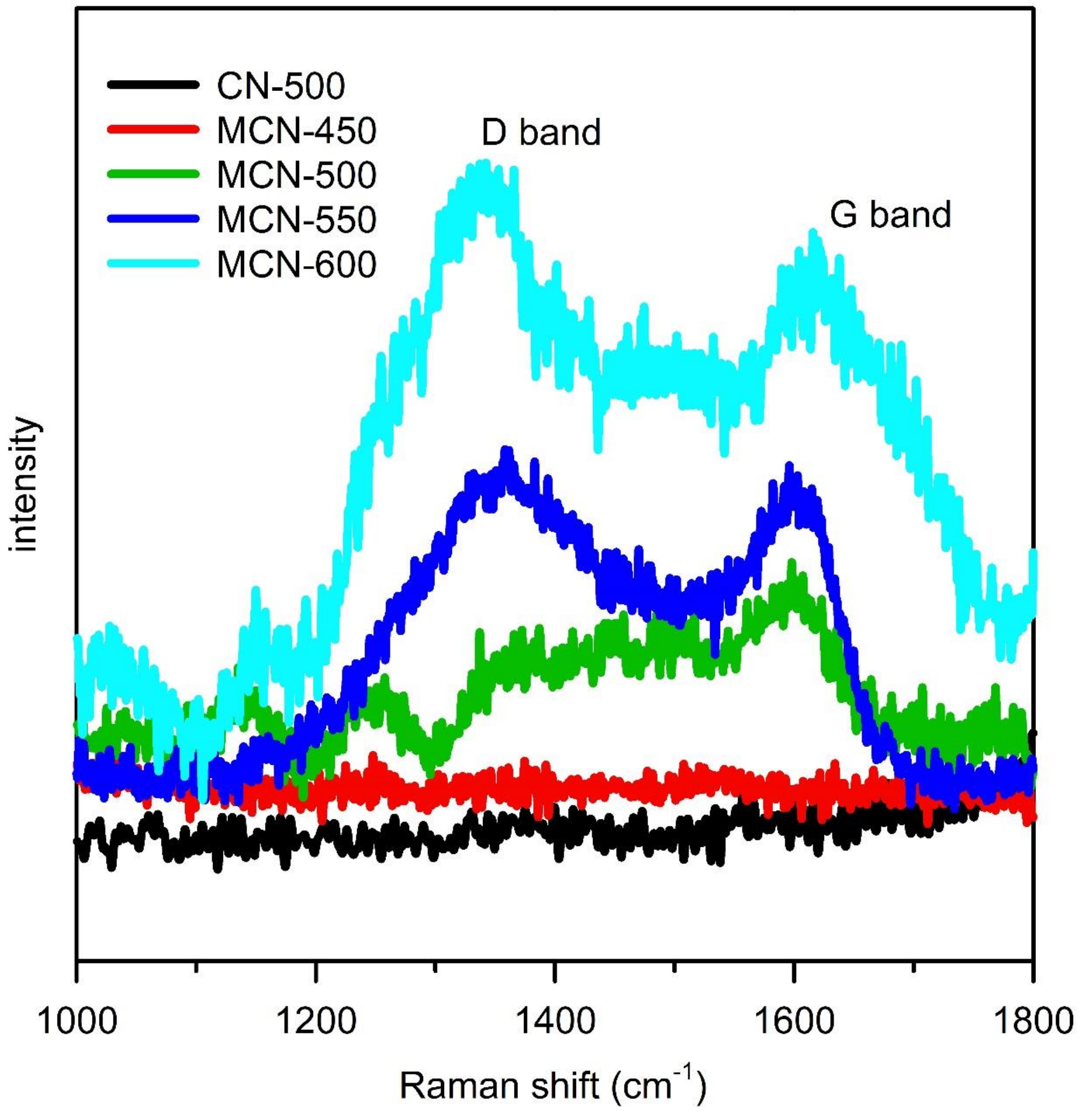

Supplementary Figure 3. Raman spectra of CN-500 and MCN-T (T = 450, 500, 550, and 600) in range of 1000 – 1800 cm^{-1}.

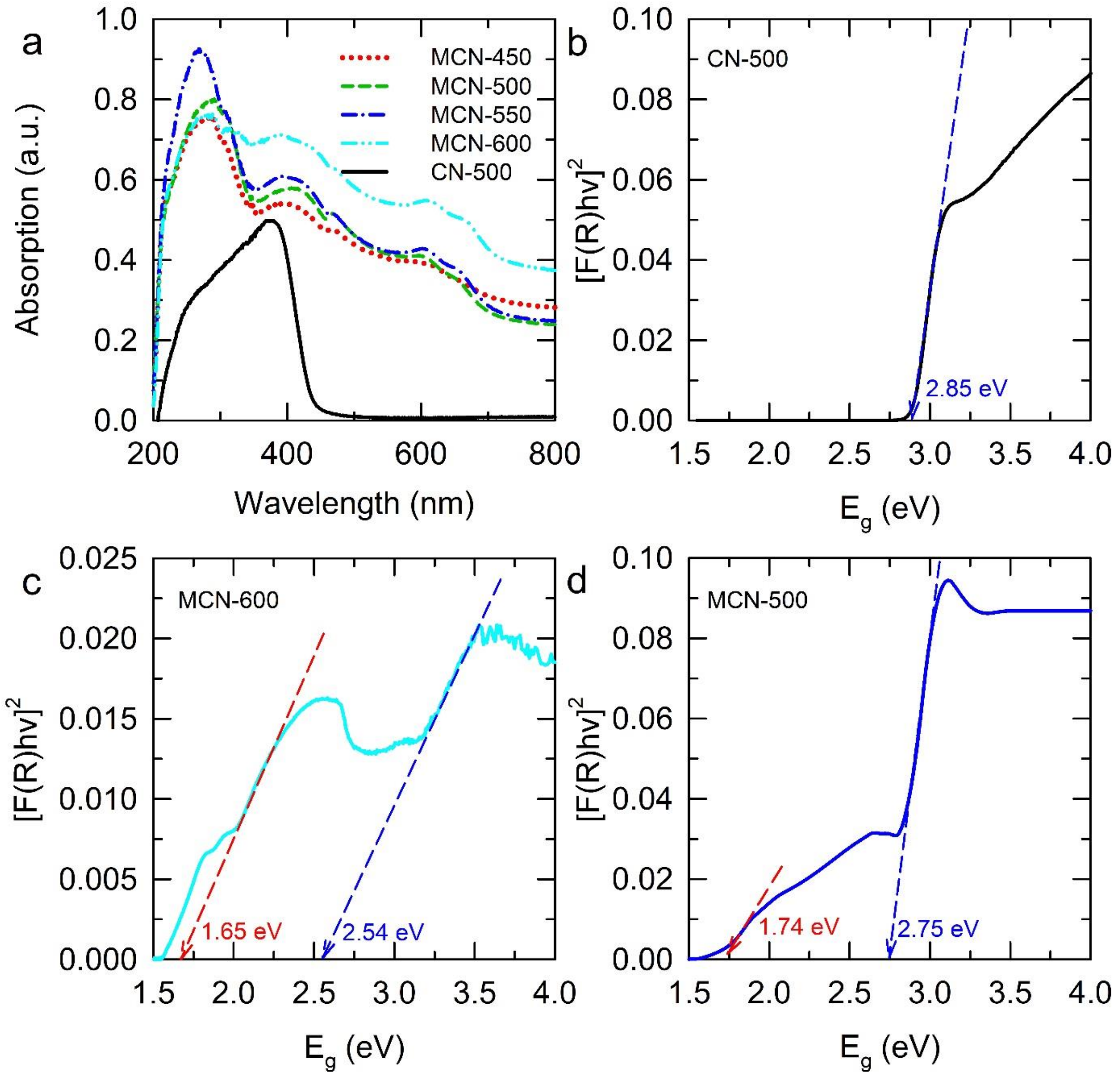

Supplementary Figure 4. (a) UV-Vis diffuse reflection spectra (DRS) of CN-500 and MCN-T (T = 450, 500, 550, and 600); Tauc plots (b) CN-500, (c) MCN-600, and (d) MCN-500 present relationship of $[F(R)h\nu]^2$ *vs*. photon energy derived from Kubelka – Munk equation: $[F(R)h\nu]^{1/n} = A(h\nu\text{-}Eg)$, in which F(R), ν, h, E_g and A are absorption coefficient, light frequency, Planck constant, band gap value and a constant. The n in Kubelka – Munk is characteristic for the transition of semiconductor: n = ½ for direct and n = 2 for indirect band gap.

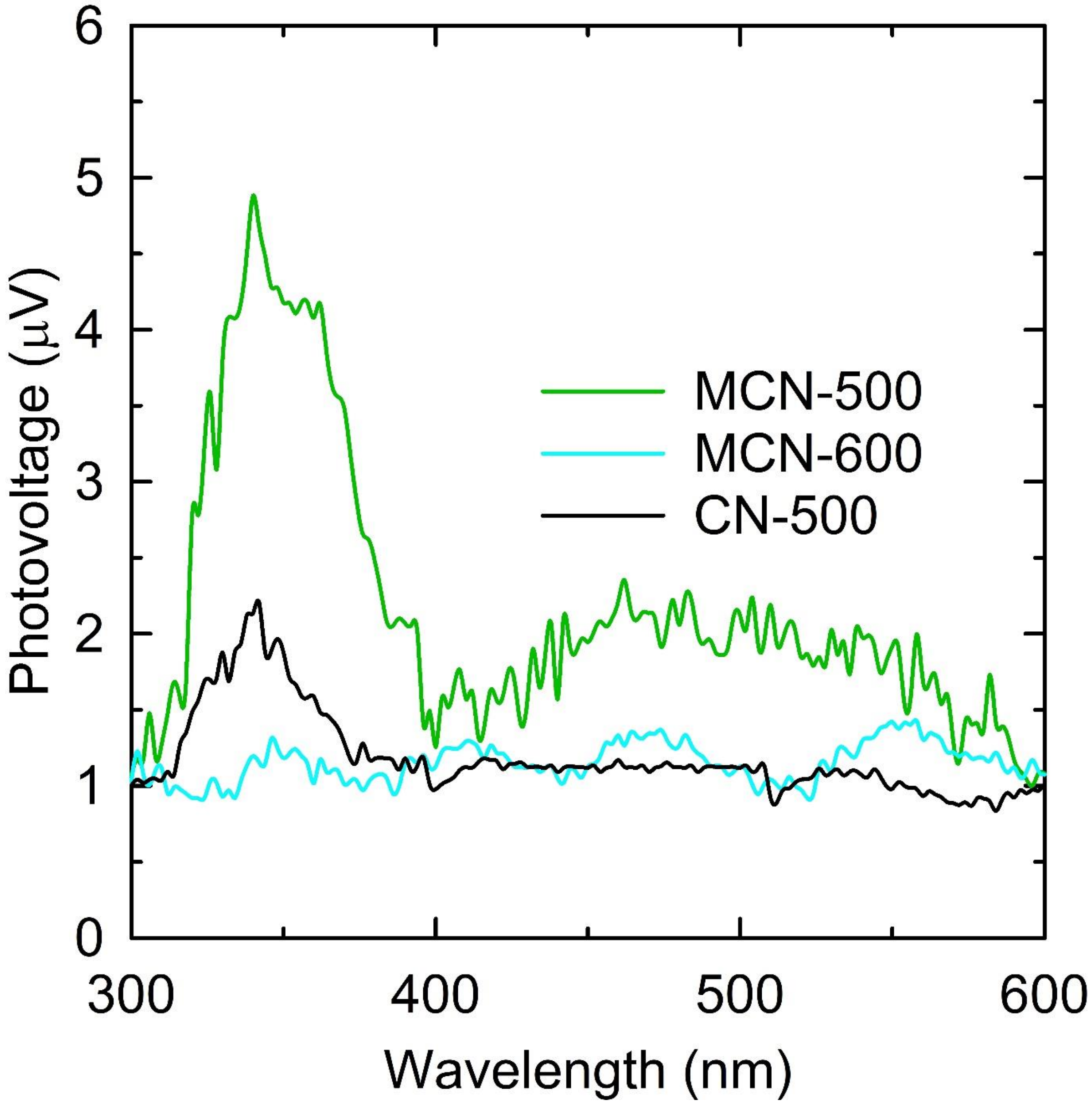

Supplementary Figure 5. Surface photovoltage spectra of MCN-500, MCN-600, and CN-500.

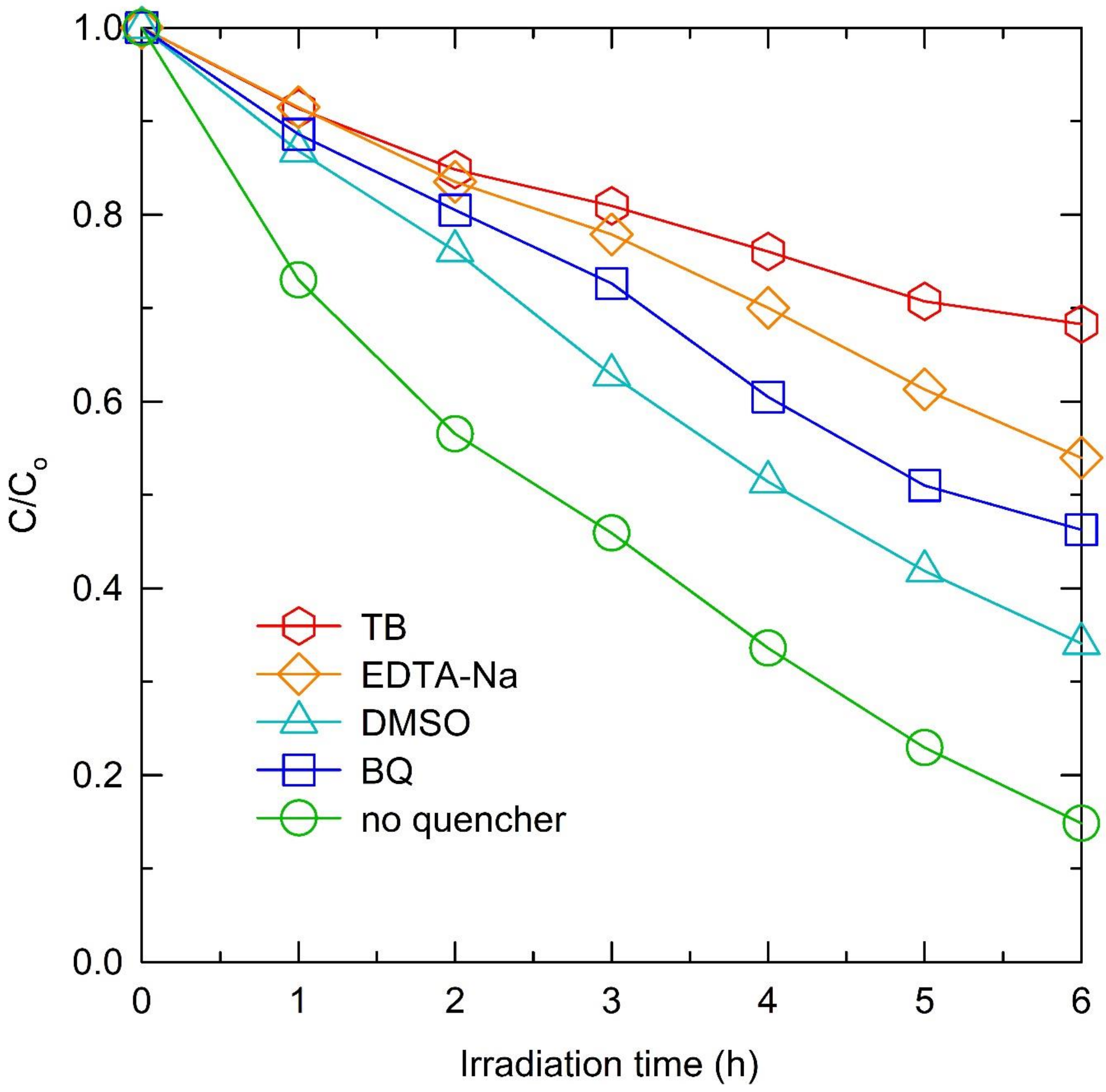

Supplementary Figure 6. Quenching effect on photocatalytic activity of MCN-500 with benzoquinone (BQ), dimethyl sulfoxide (DMSO), sodium salt of ethylenediaminetertraacetic (EDTA-Na), and tert-butanol (TB) as quenchers.

Supplemental Table 1. Comparison on photocatalytic activity of g-C_3N_4/MoS_2 based composites. ([a]RhB: Rhodamine B; [b]rate constant ratios of composites over pure g-C_3N_4)

Materials	Synthesis methods	Organic pollutants/ concentration ($mg \cdot L^{-1}$)[a]	Photocatalyst concentration ($g \cdot L^{-1}$)	Light source	Degradation efficiency (%) / time (h)	Degradation rate (h^{-1}) / compared to g-C_3N_4 (time)[b]	References
g-C_3N_4/rGO	calcination	RhB/10	1	Xenon lamp 350W (>400 nm)	-	2.466/2.6	**Chin J Cat** 36 (2015) 1009–1016
g-C_3N_4/CNTs	water bath	RhB/10	0.2	Xenon lamp 300W (>420 nm)	75.86/1	3.06/1.75	**Sep Purif Technol** 244 (2020) 1166182
MoS_2/g-C_3N_4	sonochemical	RhB/5	1	Xenon lamp 300W (>420 nm)	95/1	2.16/3.27	**Energy Environ Focus**, 4 (2015) 74–81
Fe-MoS_2/g-C_3N_4	hydrothermal	RhB/20	0.6	Xenon lamp 300W (>420 nm)	98.2/2	1.92/4.6	**ACS Sustainable Chem. Eng.** 4 (2016) 7 4055–4063
GO/MoS_2/g-C_3N_4	solvothermal	RhB/20	1	-	96.7/5	-	**ChemistrySelect** 4 (2019) 7123 – 7133
MoS_2/g-C_3N_4	calcination	RhB/10	0.2	Xenon lamp 500W (>420 nm)	-	1.53/6	**R Soc Open Sci** 5 (2018) 180187
MoS_2/g-C_3N_4	ball milling	RhB/5	0.25	-	-	0.1012/1.25	**Sci Rep** 7 (2017) 43055
MoS_2/g-C_3N_4/PAN	electrospining	RhB/10	0.5	Xenon lamp 300W (>420 nm)	50/2	0.906/1.4	**Mater Sci Semicond Process** 121 (2021) 105414
MoS_2/g-C_3N_4	calcination	RhB/30	0.625	100 W incandescent lamp (> 400 nm)	90/7	0.228/2	**This work**
